# The Association of Elevated Depression Levels and Life's Essential 8 on Cardiovascular Health With Predicted Machine Learning Models and Interpretations: Evidence From NHANES 2007–2018

**DOI:** 10.1155/da/8865176

**Published:** 2025-04-10

**Authors:** Zhixing Wu, Pengyuan Xu, Yali Zhai, Jinli Mahe, Kai Guo, Wuraola Olawole, Jiahao Zhu, Jin Han, Guannan Bai, Lin Zhang

**Affiliations:** ^1^School of Public Health and Preventive Medicine, Monash University, Melbourne, Australia; ^2^Mailman School of Public Health (Biostatistics Track), Columbia University, New York, USA; ^3^School of Engineering, Monash University, Melbourne, Australia; ^4^School of Public Health and Baotou Medical College, Inner Mongolia University of Science and Technology, Inner Mongolia, China; ^5^School of Nursing, Johns Hopkins University, Baltimore, USA; ^6^Department of Outpatient Chemotherapy, Harbin Medical University Affiliated Hospital, Harbin, China; ^7^Division of Arts and Sciences and Center for Global Health Equity, New York University Shanghai, Shanghai, China; ^8^Black Dog Institute, University of New South Wales, Sydney, Australia; ^9^Children's Hospital, Zhejiang University School of Medicine, Hangzhou, China; ^10^Suzhou Industrial Park Monash Research Institute of Science and Technology, Monash University, Suzhou, China

**Keywords:** age-specific analysis, cardiovascular health, cardiovascular risks, depression, depressive symptoms, Life's Essential 8, lifestyle patterns, machine learning algorithm, predictive and association models, psychological health

## Abstract

**Background and Objective:** The association between depression severity and cardiovascular health (CVH) represented by Life's Essential 8 (LE8) was analyzed, with a novel focus on ranked levels and different ages. Machine learning (ML) algorithms were also selected aimed at providing predictions to suggest practical recommendations for public awareness and clinical treatment.

**Methods:** We included 21,279 eligible participants from the National Health and Nutrition Examination Survey (NHANES) 2007–2018. Weighted ordinal logistic regression (LR) was utilized with further sensitivity and dose–response analysis, and ML algorithms were analyzed with SHapley Additive exPlanations (SHAP) applied to make interpretable results and visualization.

**Results:** Our studies demonstrated an inverse relationship between LE8 and elevated depressive levels, with robustness confirmed through subgroup and interaction analysis. Age-specific findings revealed middle-aged and older adults (aged 40–60 and over 60) which showed higher depresion severity, highlighting the need for greater awareness and targeted interventions. Eight ML algorithms were selected to provide predictive results, and further SHAP would become ideal supplement to increase model interpretability.

**Conclusions:** Our studies demonstrated a negative association between LE8 and elevated depressive levels and provided a suite of ML predictive models, which would generate recommendations toward clinical implications and subjective interventions.

## 1. Introduction

It has been revealed that heart disease has become the leading cause of death for the majority of people with one person dying every 33 s from cardiovascular disease (CVD) based on American statistics [1, 2]. Great attention has been aroused with ~252.2 billion dollars in year 2019 spent on healthcare service, medicine, and lost productivity due to heart diseases [3]. In order to focus on cardiovascular health (CVH) to maintain great health conditions, the American Heart Association (AHA) has introduced a scoring metric called Life's Essential 7 (LE7) with consideration of health behavior elements of diet, nicotine exposure, and physical activities as well as health factor elements of blood pressure, blood glucose, blood lipids, and body mass index (BMI) [4, 5]. Subsequently, an improved method called Life's Essential 8 (LE8) with the addition of sleep variable in 2018 for its effects in the determination of cardiovascular-related risky factors [6, 7] and these eight subindicators would comprehensively reflect such lifestyle-related and clinical aspects toward CVH [8, 9].

Numerous findings from the United States and international studies have demonstrated a consistent protective relationship between ideal CVH and several clinical and preclinical conditions [10], involving premature all-cause mortality, CVD mortality, heart failure (HF), cognitive decline, stroke, etc. For instance, the risk of incident HF was proven to be 61% lower for those with more than four ideal CVH metrics than those with only 0–2 ideal metrics for the black population [10]. Similarly, the negative association was illustrated for LE8 scoring metrics with the presence of nonalcoholic fatty liver disease (NAFLD) [11, 12], and specific LE7 elements of smoking, BMI, physical activities, blood pressure, and blood glucose were statistically associated with risk of chronic kidney disease (CKD) [13], where the impairment severity was measured by an eGFR < 60 mL/min per m^2^, identified as GFR categories of G3a (mildly to moderately decreased) to G5 (kidney failure) based on the latest 2024 KDIGO [14]. Furthermore, the negative correlation between advanced LE8 metrics and both all-cause and CVD-specific mortality was proven with the application of different databases of the National Health and Nutrition Examination Survey (NHANES), UK Biobank, and specific Kailuan prospective cohort studies accordingly [15–17].

Moreover, in terms of mental health aspects, Boehm et al. [18] have illustrated the psychological well-being's positive correlation with favorable CVH (FCVH) especially for the geriatrics, while the psychosocial discomfort including depressive symptoms was found to be negatively associated with CVH conditions [19, 20]. The scientific statement from the AHA has provided evidence for clear association between psychological health and CVD [21], and the potential role that psychological elements have played in terms of biological or behavioral processes affecting CVD was emphasized as well [21–23]. Additionally, positive psychosocial factors (PPFs) including happiness, satisfaction with life, and social support were found to be linked to CAD risks according to the UK Biobank database [24], while a large number of studies [25–28] based on NHANES have achieved similar conclusions demonstrating the inverse correlation between unfavorable CVH and decreased depressive severity.

For our research purposes, we have narrowed the psychological aspects to depression-specific factors, utilizing both elevated depressive severity and numerical measuring scores instead of previously widely applied binomial levels for the occurrence of disease [26, 27, 29]. Apart from that, we have applied LE8 metrics rather than LE7 to provide a more comprehensive landscape, and participants aged 18–20 were added to make comparison across different age brackets [9], where the majority of studies had their focus on adults with 20 years or older. In the third place, our paper would not only conduct traditional statistical association analysis but also attempt to make predictable estimates and visualized interpretations based on machine learning (ML) algorithms [30, 31], which seemed to be innovative study areas and helpful to clinical diagnosis and treatment processes in the long term.

## 2. Materials and Methods

### 2.1. Study Population

Our research was based on the cross-sectional and national-wide survey called the NHANES conducted by the National Center for Health Statistics (NCHS) as part of the Centers for Disease Control and Prevention (CDC). A complex, stratified, and multistage probability sampling design was utilized for national health and nutritional data collection in the United States during 2-year sampling cycles [32].

Among all the 59,842 observations spanning from NHANES 2007–2018, we have excluded those participants aged <18 years old without depression records and pregnant individuals. For the remaining 36,208 participants, nonapplicable observations from LE8 elements (*n* = 11,878) and depression variables (*n* = 251) were out of scope. Accounting for demographic and socioeconomic variables of age, gender, race, education level, marital status, household size, and family income-to-poverty ratio, our final analytical sample included 22,369 eligible participants, including 21,279 adults over the age of 20 years old and 1090 young people aged 18–20. The detailed selection criteria and exclusion flowchart would be shown in Figure S1.

### 2.2. Measurements and Variables

With the purpose of quantitative evaluating CVH, the AHA has developed the scoring metrics called LE8 consisting of four health behavior variables of diet, physical activity, nicotine exposure, and sleep health as well as four health factor elements of BMI, blood lipids, blood glucose, and blood pressure. Scoring metrics were differently applied to adults aged ≥20 years old and young participants (<20 years old). To support a composite and aggregate score, unweighted score averages were computed with each component assigned a quantitative score between 0 and 100 points. Detailed threshold values were advised by AHA's guidelines when determining the overall CVH categories (high CVH, LE8 score ≥80; moderate CVH, 50 ≤ LE8 score <80; and low CVH, LE8 score <50). See metric scoring and quantifying details in Table S1.

Depression variables were defined and calculated according to the Patient Health Questionnaire (PHQ-9) with quantitative measurement and diagnosis of depression severity [33]. The nine-item instrument was designed to evaluate the overall impairment of symptoms based on DSM-IV depression diagnostic criteria [34]. Trained interviewers administered the questions at the mobile examination center (MEC) using the computer-assisted personal interview (CAPI) system as part of the MEC interview process [35]. The questions specifically focused on assessing the frequency of depression symptoms experienced over the past 2 weeks: not at all (0 point), several days (1 point), more than half the days (2 points), and nearly every day (3 points). In total, the depression scores were calculated from 0 to 27 points with higher marks representing more severe symptoms. The classification rule would be as follows, minimal (0–4 points), mild (5–9 points), moderate (10–14 points), moderately severe (15–19 points), and severe (20–27 points), and the group having score of 10 points or above could be denoted as depression disease accordingly [34, 36].

Based on our study design, age was segmented for adults (≥20 years old) and teenagers (18–20 years old), while adults were subdivided into three age groups (20–40, 40−60, and ≥60), respectively. All seven covariates were analyzed with adults' characteristics: gender (male or female), age (20–40, 40−60, and ≥60), race or ethnicity (Mexican American, non-Hispanic White, non-Hispanic Black, and other), education (less than high school, high school or equivalent, and college or above), marital (married, never married, and other), family poverty income ratio (PIR) with cutoff values of 1.3 and 3.5, and family household sizes (1–3, 3–5, and more than 5 members). While for teenagers whose records might be lacking in family related items, features of gender and race were summarized as well.

### 2.3. Statistical Analysis

With the purpose of being objective and representative, sample weight adjustment was considered based on NHANES with complex survey designs. Six periodic survey cycles were analyzed according to NHANES guidelines.

Firstly, baseline characteristics for adults and teenagers were summarized with stratification of low, moderate, and high LE8. Both sample number (*n*) and weighted percentage (%) were illustrated for categorical variables, and weighted Rao-Scott chi-squared tests were utilized for comparisons. Then depression-related profiles were presented within four age groups across three LE8 subgroups. The mean values and 95% confidence intervals (CIs) were shown for continuous depression scores, while counts and percentages were analyzed for five depression levels and occurrence of disease.

In order to generate statistical relationship between elevated depression levels and LE8 divisions, weighted ordinal logistic regression (LR) models were chosen accordingly. Odds ratios (ORs) were calculated in the crude model, the age-adjusted model, and the new model considering all seven covariates of age, gender, race, education, marital, PIR, and household sizes. Furthermore, forest plots along with corresponding sensitivity tests have validated the model robustness with all possible stratified subgroups, and restricted cubic spline (RCS) plots were drawn to evaluate the dose–response and nonlinearity with respectively *p*-values shown.

To identify predictive and interpretable effects using CVH risk factors of LE8 variables, eight ML algorithms were applied toward depressive conditions, including eXtreme Gradient Boosting (XGBoost), decision tree (DT), LR, multilayer perception (MLP), naive Bayes (NB) classification, *K*-nearest neighbors (KNN), random forest (RF), and support vector machine (SVM). Corresponding receiver operating characteristic (ROC) curves and the area under the curve (AUC) statistics were investigated and four indicators of accuracy, recall, precision, and F1 scores along with potential sensitivity or specificity calculated. The whole dataset cohort was classified into a 70% training and remaining 30% validation testing cohort as well as additional cross-validation processes, and additional feature visualization, waterfall charts, and SHapley Additive exPlanations (SHAP) tools were illustrated for interpretable purposes and decision-making explanations.

All analysis and computations were done based on R software, and a suite of packages within Tidymodels were applied for ML development. For our analysis, all *p*-values have 0.05 as the threshold for statistical significance.

## 3. Results

### 3.1. Baseline Characteristics of the Study Participants

Among all 21,279 eligible adults for our studies, over 60% (65.29%) belonged to the moderate LE8, and around 18.31% and 16.39% participants were labeled as low or high LE8. Taking overall LE8 status into consideration, the majority of demographics include 51.1% of female, 37.7% with ages from 40 to 60, 68.9% being non-Hispanic White, 63.4% having college or above background, 55.6% identified as married, 44.2% showing family-wise PIR greater than 3.5, and 49.6% with living environment of one to three members. Focusing on teenagers aged 18 or 19, about 52.5% were male, and the non-Hispanic White also ranked the first (55.1%) for the overall LE8 scores.

With stratification of LE8 levels, high LE8 (LE8 scores ≥80) has consisted of 58.8% of females, 48.6% of young adults aged 20–40 years old, 74.6% of non-Hispanic White, 59.3% of married citizens, and the majority belonging to family PIR ratio larger than 3.5 and household sizes of one to three members. In terms of different age brackets, around 20% of teenagers with 18–19 years old were regarded as high LE8, and such percentage has climbed to about 48.6% for adults aged 20–40, with further gradual decline to 32.8% for 40–60 years old and final approximation of 18.6% for the elderly participants. See detailed statistics and illustrations in Table 1. Weighted survey designs were conducted separately for the total, low, moderate, and high LE8 categories, with stratification based on different covariates. Proportional percentages for each subgroup were calculated, ensuring that the total sum in each LE8 group equaled 100%. The majority of eligible participants in total category tend to have college or above education (63.4%), with relative lower figure in low LE8 but higher percent of 84.4% for high LE8 group, potentially showing relative prevalence of well-educated people in favorable CVH groups. Similarly for family PIR over 3.5, 44.2% of total participants belonged to such group while that proportion rose to 59.7% for high LE8.

Overall information about depression situations were summarized in Table S2 across three LE8 subgroups. It was revealed that people aged 40–60 tend to have worse depression indicators compared with other three age categories, with the highest mean depression value of 3.28 (95% CI, 3.097–3.459), largest percent of 1.1% regarding severe depression levels as well as around 9.2% of participants diagnosed with depression diseases compared with corresponding percent of 6.4%, 8.3%, and 6.4%, for people with age 18–20, 20−40, over 60 years old. Moreover, it was noticeable that numerical depression scores have shown a dropping pattern with elevated LE8 levels, which was proven to be consistent among all age subdivisions as well.

### 3.2. Relationship Between LE8 and Elevated Depression Categories

According to the crude model, the age-adjusted model, and new model considering all seven covariates, an inverse relationship between CVH measured by three LE8 categories and ranked depression levels was represented. Specifically, the adjusted OR for moderate and high LE8 with reference of low LE8 would be 0.447 (95% CI, 0.400–0.500) and 0.231 (95% CI, 0.196–0.273), meaning the odds of being more likely to be classified into worse depression levels were 55.3% lower for moderate CVH than low CVH and even 76.9% lower for High CVH compared with low CVH. Taking numerical LE8 scores as an example, with one unit increase in overall LE8 score, health behavior score and health factor score would correspond to reducing odds of 3.3% (OR, 0.967; 95% CI, 0.964–0.970), 2.5% (OR, 0.975; 95% CI, 0.973, 0.977) and 1.0% (OR, 0.990; 95% CI, 0.987–0.993) with all seven covariates adjusted. Details were shown in Table 2 with almost all statistical significance shown by *p*-values smaller than 0.05.

As was presented in Table S3, such negative association between LE8 as well as scores and elevated depression levels could still hold for all age groups. Focusing on specific LE8 subindicative scores including health behavior or factor items, sleep seemed significant for three groups older than 20 years old with relatively smaller OR values, representing larger changing scale about odds for depression levels with comparison of other LE8 variables. However, such phenomenon was not evident for teens aged 18–20, and diet and blood pressure seemed meaningful variables with smaller OR values for these young adults.

In order to concentrate on the CVH conditions of different age groups, we presented detailed demographic summaries for adults aged 20–40, 40−60, and over 60 years old in Table S4 and made comparisons for all age brackets in Table S5. Specifically, CVH tend to have worse conditions along with the increases in ages, with middle-aged or elderly accounting for the majority (40.14% and 37.45%, respectively) of low LE8 categories and adults over 60 constituting 34.75% of the moderate LE8 levels while the young adults between 20 and 40 years old having the highest percentage (45.36%) in high LE8 status.

### 3.3. Sensitivity Analysis and RCS Plots

**Figure 1:**
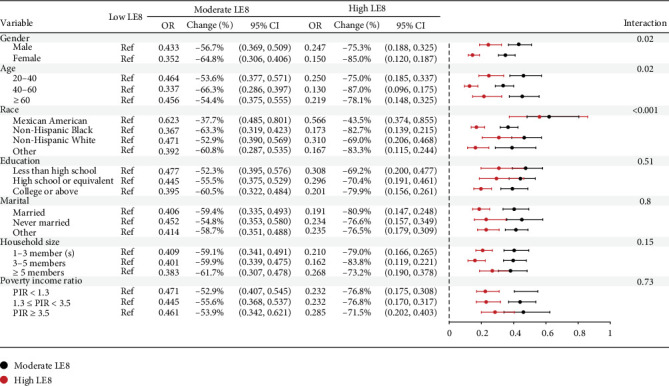
Sensitivity analysis between LE8 and elevated depression levels.

Sensitivity analysis has been conducted with stratification of all seven covariates for adults aged over 20 years old with low LE8 subgroup denoted as the reference group. According to Figure 1, the inverse relationship between LE8 and ranked depression conditions was consistent across all subgroups with all calculated OR smaller than one. More specifically, such negative association was apparently stronger in subgroups of female, people between 40 and 60, educational background of college or above, and married status. For age-specific items, changing from low to moderate and high LE8 groups has corresponded to OR changed from 0.464 (95% CI, 0.377–0.571) to 0.250 (95% CI, 0.185–0.337) for young adults aged 20–40, together with OR from 0.456 (95% CI, 0.375–0.555) to 0.219 (95% CI, 0.148–0.325) for people over 60. For middle-aged people aged 40–60, the odds of being more likely to fall into severe depression conditions were reduced by 66.3% for moderate LE8 and even dropped by ~87% for high LE8 with comparisons of low LE8 teams. Further interaction check has also been conducted, and almost all interactive terms were proven to be insignificant due to *p*-values larger than 0.05.

Moreover, separate RCS plots were presented to identify specific trends with four age brackets. The overall negative dose–response relationship between LE8 and depression levels was demonstrated due to all significant overall *p*-values. While for nonlinearity check, participants older than 20 years old had corresponding *p*-values smaller than cutoff value of 0.01 with group with 18–20 ages group failing the test accordingly. See details in Figure S2 for graphical explanations.

### 3.4. ML Predictive Models and Representative Interpretations

**Figure 2:**
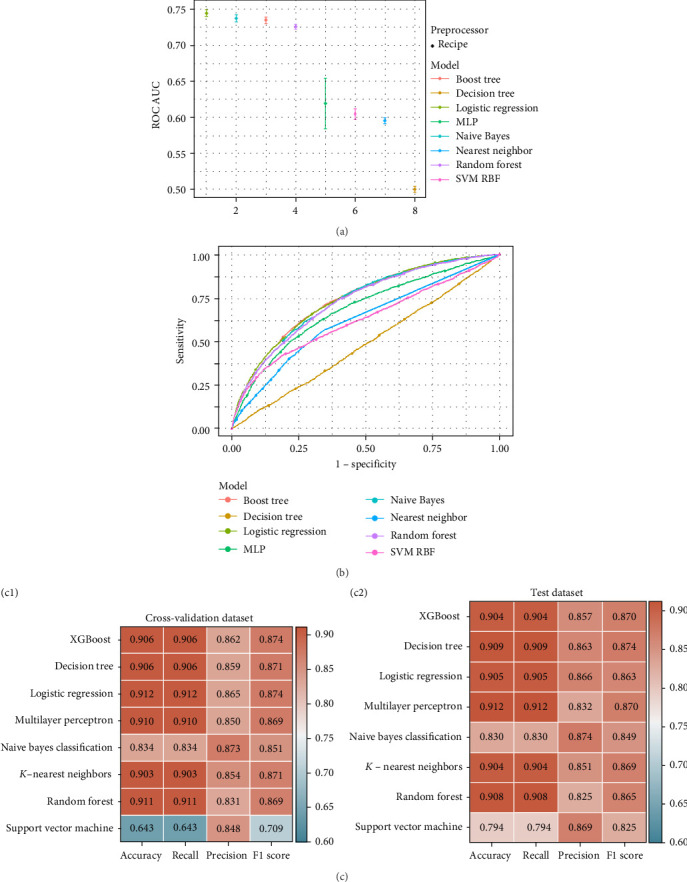
Performance comparison of machine learning models: (A) error bars for eight models in cohort; (B) ROC curves in terms of sensitivity and specificity; and (C) performance of eight models measured by accuracy, recall, precision, and F1 score for cross-validation dataset (c1) and test dataset (c2).

As could be vividly shown in Figure 2B, eight ML models were generated with AUC values all larger than or equal to 0.5. LR model has shown the greatest AUC of 0.745 with NB model following closely with figure of 0.737, and the XGBoost model ranked the third position with AUC equaling to 0.735. Moreover, different error bars were presented in Figure 2A, and in total, four algorithms models, namely, LR, NB, XGBoost, and RF, were found to present the best fitted predicted results with cutoff AUC number of 0.7. Furthermore, four indicative elements of accuracy, recall, precision, and F1 score were summarized in Figure 2C to present comprehensive selection criteria for optimal selection, and the whole ML package involving all eight models would be of suitability in terms of predictive purposes based on corresponding simulation and validation conducted.

Apart from that, representative interpretations using SHAP values based on XGBoost model were presented according to primary features related to CVH scores and subindicators. As was concluded from Figure S3A, characteristics such as gender, education, and marital would exert significance effects on predictive results with negative relation for males and married participants in contrast with positive impact for higher academic background. Based on Figure S3C,D, health behavior score, gender categories, and poverty status have become the top three significant variables with respect to impact on predictive models, and the seven elements identified in Figure S3B were proven to represent evident effects toward final prediction results.

## 4. Discussion

Based on our NHANES analysis toward cardiovascular risks represented by LE8 indicators as well as depression variables, overall statistical analysis has robustly illustrated the negative association with specified concentration on four different age brackets. Additionally, a package of eight ML models were selected to present predictive results with SHAP values applied for increasing model interpretability.

CVDs have continuously aroused people's attention due to its high prevalence and mortality, accounting for ~18 million deaths in recent years based on the World Health Organization (WHO) statistics [37]. Several prospective cohort studies discovered the negative associations between ideal LE7 and depressive symptoms according to participants established from the United States, China, France, Brazil [28, 38–40], showing the declining trend of depression risks with greater number of CVH levels. With further adoption of new LE8 scoring metrics with incorporation of sleep factor, similar negative association between LE8 and depression would also be proven [6, 7, 27].

Depression is a complex condition with diverse symptom patterns, including feelings of hopelessness or pessimism and unusual behavioral changes, such as increased alcohol or drug usage [41]. Symptoms can vary widely across individuals across age, race, gender, and cultural background, differing in type, frequency, severity, and so on [41–43]. In addition to psychological signs, depression may also present with physical manifestations, such as a racing heartbeat, chest tightness, chronic headaches, or digestive problems [41, 43]. Therefore, a comprehensive and objective quantifying instrument is of great significance with consideration various dimensions. The PHQ-9, a useful nine-item instrument currently widely applied, could not only identified as the depression screener but also validated as a continuous measure of depressive severity [33, 36]. It has been proven that PHQ-9 is acceptable for major US sociodemographic groups and subgroup comparisons by Patel et al. with scientific assessment of the factor structure and measurement invariance of PHQ-9 [44]. Such PHQ-9 administration held by trained interviewers was further proven to hold diagnostic validity in comparison with the original clinician-administered PRIME-MD but even with more efficiency in practical applications as well [34].

The majority of previous studies commonly applied binomial-dependent variables with occurrence of depression disease with a cutoff PHQ-9 score of 10 points [33, 34, 45]. Our studies, on the contrary, has the revolution to apply elevated or ranked depressive severity levels and assess relationship between ranked ordinal-dependent variables with both continuous and categorical LE8 indicators. Our interpretative results have robustly showed the greater likelihood with worse depressive severity conditions with increasing LE8 scores, regardless of age groups selected and covariates about gender, education, marital, family income, and size added.

Apart from that, another advanced investigation could be the age-specific subgroup analysis. Adult participants have aroused great attention with more mature status and thorough information [4, 5, 9] in previous studies, while our researches have not only included observations from eligible teenagers aged 18–20 whose PHQ-9 responses toward depressive symptoms were available but also studied sensitivity of associated correlation across different age groups with age 20 as a cutoff threshold. In terms of CVH conditions, comparisons across four age groups in our studies revealed that unfavorable CVH outcomes were more apparent in older population, while the majority of those with high CVH belong to young adults in 20–40 age range. This observation was consistent with several studies [46–48] highlighting that older patients tend to experience a higher prevalence of CVD and risk factors [49, 50], often accompanied by age-related comorbidities [50, 51]. For instance, a study by Lettino et al. [46] found that the prevalence of hypertension was 28% among individuals aged 20–40 but rose significantly to 77% in those over 65 [46, 52]. Similarly, the incidence of hyperlipidemia was 76% for older individuals with a mean age of 69, compared to 41% in younger ones with a mean age of 29 [53], and cognitive or emotional factors of the geriatric could influence their capabilities to adhere to lifestyle changes or pharmacological treatment to some extent [46, 51, 54].

With regard to age-specific depression analysis, our studies revealed that adults aged 40–60 had worst depression conditions with 9.2% with a diagnosis of depressive illness, in contrast to 6.4%, 8.3%, and 8.4% for individuals with age of 18–20, 20−40, and over 60 accordingly. This finding is of importance in arousing greater public awareness of depression, especially for middle aged or the elderly. According to WHO statistics and Global Health Estimates (GHE) [55, 56], around 14% of adults over 60 live with a mental disorder, accounting for 10.6% of total disability measured by disability-adjusted life years (DALYs), and those elderly are suffering from a greater risk of depression and anxiety with mental health conditions underrecognized and undertreated. The term “geriatric giants” was introduced in 1965 and evolved to encompass four new syndromes where depression has played a unignorable role for the elderly [57]. Major depression has affected 2% of adults aged 55 and older, while 10%–15% experience clinically significant depressive symptoms as well, with the corresponding prevalence increasing with age [58]. Indeed, depressed older people were more likely to exhibit cognitive impairments, somatic complaints, and diminished interest than the younger individuals [59, 60]. Therefore, with purpose of investigating depression among the geriatric patient, several instruments were put forward. One tool called Geriatric Depression Scale (GDS), including three versions of GDS-30, GDS-15, and GDS-5, was regarded as the widely applied method for depression detection toward older population rather than general population [49, 50, 61]. Another method called PHQ-9, which was implemented in our NHANES analysis as well, was also appraised for its superior reliability and validity for detecting major depression [62]. Furthermore, when examining PHQ-9 and GDS-15 in older adults aged more than 60 years, both of them achieved superior outcomes with the inner consistency rate of 96.1%, and PHQ-9 seemed more attractive due to its ease of use and relative brevity [63]. Additionally, based on comparisons across various depression screening tools with respect to older veterans, the PHQ-9, PHQ-2/PHQ-3, GDS-15, and GDS-5 were all deemed effective. Notably, GDS-15 and GDS-5 were particularly recommended for older adults in outpatient psychiatric clinics or those who are medically fragile and hospitalized, as these populations tend to have higher base rates of depression. In contrast, the PHQ-9 was advised for medically stable older individuals or those in outpatient settings without the heavy burden imposed on fully functional populations [64].

When it comes to specific LE8 scoring components, health behavior elements involving diet, exercise, smoking, and sleeping had larger OR compared with health factor variables of BMI, blood glucose, blood lipids, and blood pressure. Such findings were plausible for the closer relationship as well as direct effects between depressive mental disease and behavior changes or patterns, which were supported by studies like Zhang et al. [28] and Zeng et al. [27] stating the robust linkage with respect to depression and health behaviors. Moreover, demographic characteristics have revealed portrait of subgroups belonging to high CVH with higher percentage of female, non-Hispanic White, college or above background, married status, family size of one to three members, and wealthier conditions (PIR above 3.5). Such phenomena would be explained by similar findings between socioeconomic status (SES) and CVD burden [65], with less advantageous background subgroups showing prevalence in disease incidence or mortality with biological, behavioral, and psychosocial factors taking effect [66, 67]. Furthermore, with age-specific considerations, sleep variable seemed to have larger scale of change in terms of depressive severity for adults over 20 compared with other seven variables. This finding was supported by analyzing relationship between short or extended sleep and depressive risky levels, and such association was proven apparent with adults and elderly particularly [68–70].

Instead of merely conducting association analysis based on statistical techniques, another improved point was generating ML models for cohort data predictions. Eight widely used ML algorithms were employed [71, 72], with four of them proven to be best fitting in our studies. Specifically, such diagnostic effects would play an important role in identifying and estimating possibility of depressive diseases based on biomarkers or features related to LE8 scoring and SES conditions at a very early stage, which seemed to be more direct and easy-to-implement with quantitative scoring metrics implying obscure and mysterious depressive challenges [30, 31]. Furthermore, SHAP tools were applied to increase the interpretability by prioritizing the features and visualizing the model's decision-making capacities [73, 74], which would generate predictable explanations for decisions and estimates in a precise manner [72, 75].

Several strengths were apparent in our studies. Firstly, representative and nationwide survey was analyzed with large-scale observations, and possible weight adjustment enabled extension and estimation to population perspectives as well. Secondly, advanced LE8 metrics replaced previous LE7, and four age brackets especially the young adults aged 18–20 were included as a supplement. Thirdly, statistical analysis was improved by utilizing ordinal regression model to analyze elevated or ranked data instead of common binomial responses, and further predictable ML models together with SHAP values and feature diagrams were added which would contribute to clinical diagnosis procedures.

However, some limitations could not be ignored. Since subjective answers from questionnaires were collected, memory distortion and recall bias related to diet, physical activity, smoking, and sleeping might be inevitable. And such cross-sectional study instead of prospective cohorts failed to imply causal relationships, and other covariates or compounders might be omitted from our selected variables. Thirdly, while PHQ-9 is widely used due to its effectiveness and convenience, it is important to acknowledge that certain atypical symptoms of depression may go unnoticed, and some physical discomforts might be overlooked as well. And given that PHQ-9 in NHANES dataset could make assessment over a fixed 2-week period, episodic patterns or seasonal affective disorder (SAD) might not be captured. Therefore, more advanced instruments would be called for with inclusion of mental or physical, common, or unusual symptoms, along with interviews with wider research window such as the similar Canadian Community Health Survey conducted using PHQ-9 throughout the whole year [76]. Additionally in terms of ML predictive models, the lack of adaptive and automatic hyperparameter tuning process might become problematic, and manual hyperparameter adjustment and extensive experimentation was time-consuming and computationally expensive to some extent.

## 5. Conclusion

In conclusion, a negative relationship between CVH measured by LE8 and elevated depressive severity was demonstrated, and further subgroup stratification and sensitivity check have proven the model robustness. Four age groups with inclusion of teenagers below 20 were classified to make comparisons, and sleep variable was found to have more sensitive effects toward depression in adults aged 20 or above. Participants with profiles of less advantageous SES conditions had worse depressive symptoms, and a package of ML models were identified to make predictable results with SHAP tools providing assistance in clinical diagnosis and treatment.

## Figures and Tables

**Table 1:** Demographic and socioeconomic characteristics of the study participants with LE8 classifications.

Adult (age ≥ 20)									
Characteristics	Total		Low LE8		Moderate LE8		High LE8		*p*-Value
Sample, *n*	21,279		3897 (18.31%)		13,894 (65.29%)		3488 (16.39%)		
	Sample	Pct.	Sample	Pct.	Sample	Pct.	Sample	Pct.	
Gender, *n* (%)									<0.001
Male	10,405	48.9%	2074	53.2%	7003	50.4%	1439	41.3%	—
Female	10,874	51.1%	1823	46.8%	6891	49.6%	2049	58.8%	—
Age, *n* (%)									<0.001
20–40	7575	35.6%	944	24.2%	4749	34.2%	1696	48.6%	—
40–60	8022	37.7%	1796	46.1%	5185	37.3%	1143	32.8%	—
≥60	5681	26.7%	1157	29.7%	3960	28.5%	649	18.6%	—
Race, *n* (%)									<0.001
Mexican American	1758	8.3%	331	8.5%	1222	8.8%	223	6.4%	—
Non-Hispanic Black	2136	10.0%	613	15.7%	1457	10.5%	157	4.5%	—
Non-Hispanic White	14,657	68.9%	2539	65.1%	9438	67.9%	2601	74.6%	—
Other	2726	12.8%	415	10.6%	1776	12.8%	507	14.5%	—
Education, *n* (%)									<0.001
Less than high school	2915	13.7%	977	25.1%	1939	14.0%	153	4.4%	—
High school or equivalent	4873	22.9%	1278	32.8%	3376	24.3%	391	11.2%	—
College or above	13,491	63.4%	1642	42.1%	8578	61.7%	2944	84.4%	—
Marital, *n* (%)									<0.001
Married	11,831	55.6%	1915	49.1%	7775	56.0%	2068	59.3%	—
Never married	3937	18.5%	569	14.6%	2441	17.6%	844	24.2%	—
Other	5511	25.9%	1413	36.3%	3678	26.5%	576	16.5%	—
Family PIR, *n* (%)									<0.001
PIR < 1.3	4447	20.9%	1321	33.9%	2862	20.6%	424	12.2%	—
1.3 ≤ PIR < 3.5	7426	34.9%	1520	39.0%	5017	36.1%	982	28.1%	—
PIR ≥ 3.5	9405	44.2%	1056	27.1%	6013	43.3%	2082	59.7%	—
Household size, *n* (%)									0.1
1–3 member(s)	10,554	49.6%	1947	50.0%	6884	49.6%	1732	49.7%	—
3–5 members	7384	34.7%	1268	32.6%	4823	34.7%	1262	36.2%	—
≥5 members	3341	15.7%	682	17.5%	2187	15.7%	493	14.1%	—

**Teen (age 18–20)**									
**Characteristics**	**Total**		**Low LE8**		**Moderate LE8**		**High LE8**		***p*-Value**
**Sample, *n***	**1090**		**84 (7.71%)**		**773 (70.92%)**		**233 (21.38%)**		
	**Sample**	**Pct.**	**Sample**	**Pct.**	**Sample**	**Pct.**	**Sample**	**Pct.**	

Gender, *n* (%)									0.9
Male	572	52.5%	46	55.2%	404	52.3%	122	52.4%	—
Female	518	47.5%	38	44.8%	369	47.7%	111	47.6%	—
Race, *n* (%)									0.02
Mexican American	189	17.3%	18	21.2%	138	17.8%	34	14.8%	—
Non-Hispanic Black	159	14.6%	18	20.9%	124	16.0%	22	9.3%	—
Non-Hispanic White	601	55.1%	43	51.4%	397	51.3%	153	65.7%	—
Other	142	13.0%	5	6.5%	114	14.8%	24	10.3%	—

*Note:* Marital status labeled as “Other” includes separated, widowed, divorced, and living with partner.

Abbreviations: LE8, Life's Essential 8; Pct., percentage; PIR, poverty–icome ratio.

**Table 2:** Weighted ordinal logistic regression representing the relationship for adults over 20 years old between LE8 and elevated depression categories.

Variables	Crude model					Model adjusted for age						New model			
	OR	Change	95% CI	*p*-Value		OR	Change	95% CI			*p*-Value	OR	Change	95% CI	*p*-Value
LE8 category															
Low LE8	1 (reference)					1 (reference)						1 (reference)			
Moderate LE8	0.403	−59.8%	(0.361, 0.449)		<0.001	0.396	−60.4%		(0.355, 0.442)	<0.001		0.447	−55.3%	(0.400, 0.500)	<0.001
High LE8	0.200	−80.0%	(0.170, 0.236)		<0.001	0.189	−81.1%		(0.161, 0.223)	<0.001		0.231	−76.9%	(0.196, 0.273)	<0.001
LE8 scores															
Total score	0.965	−3.5%	(0.962, 0.968)		<0.001	0.964	−3.6%		(0.961, 0.966)	<0.001		0.967	−3.3%	(0.964, 0.970)	<0.001
Health behavior score	0.972	−2.8%	(0.970, 0.974)		<0.001	0.972	−2.8%		(0.970, 0.974)	<0.001		0.975	−2.5%	(0.973, 0.977)	<0.001
Diet	0.992	−0.8%	(0.990, 0.993)		<0.001	0.992	−0.8%		(0.990, 0.993)	<0.001		0.993	−0.7%	(0.992, 0.995)	<0.001
Exercise	0.992	−0.8%	(0.991, 0.993)		<0.001	0.992	−0.8%		(0.991, 0.993)	<0.001		0.994	−0.6%	(0.993, 0.995)	<0.001
Smoke	0.990	−1.0%	(0.989, 0.991)		<0.001	0.990	−1.0%		(0.989, 0.992)	<0.001		0.992	−0.8%	(0.991, 0.993)	<0.001
Sleep	0.982	−1.8%	(0.980, 0.985)		<0.001	0.983	−1.8%		(0.981, 0.985)	<0.001		0.985	−1.5%	(0.983, 0.987)	<0.001
Health factor score	0.991	−0.9%	(0.988, 0.993)		<0.001	0.989	−1.1%		(0.986, 0.991)	<0.001		0.990	−1.0%	(0.987, 0.993)	<0.001
BMI	0.994	−0.6%	(0.992, 0.995)		<0.001	0.994	−0.6%		(0.992, 0.995)	<0.001		0.994	−0.6%	(0.992, 0.996)	<0.001
Blood lipids	0.997	−0.3%	(0.996, 0.999)		<0.001	0.997	−0.3%		(0.996, 0.999)	<0.001		0.997	−0.3%	(0.996, 0.999)	<0.001
Blood glucose	0.996	−0.4%	(0.994, 0.998)		<0.001	0.994	−0.6%		(0.992, 0.996)	<0.001		0.996	−0.4%	(0.994, 0.998)	<0.001
Blood pressure	0.999	−0.1%	(0.998, 1.001)		0.479	0.999	−0.1%		(0.997, 1.000)	0.122		0.999	−0.1%	(0.997, 1.001)	0.297

*Note:* Crude model: Consider LE8-related variables without further adjustment. Model adjusted for age: Crude model plus adjusted age variables. New model: Crude model plus all seven covariates adjusted for age, gender, gender, education, marital, poverty–income ratio and household sizes.

Abbreviations: BMI, body mass index; CI, confidence interval; LE8, Life's Essential 8; OR, odds ratio.

## Data Availability

This study utilized data from the NHANES open database. The NHANES dataset can be accessed online, and access is permitted openly: https://wwwn.cdc.gov/nchs/nhanes/Default.aspx.

## Nomenclature

AHA: American Heart Association
AUC: Area under the curve
BMI: Body mass index
CAPI: Computer-assisted personal interview
CDC: Centers for Disease Control and Prevention
CIs: Confidence intervals
CKD: Chronic kidney disease
CVD: Cardiovascular disease
CVH: Cardiovascular health
DALYs: Disability-adjusted life years
DT: Decision tree
FCVH: Favorable cardiovascular health
GDS: Geriatric Depression Scale
GHE: Global health estimates
HF: Heart failure
KNN: *K*-nearest neighbors
LE7: Life's Essential 7
LE8: Life's Essential 8
LR: Logistic regression
MEC: Mobile examination center
ML: Machine learning
MLP: Multilayer perception
NAFLD: Nonalcoholic fatty liver disease
NB: Naive Bayes
NCHS: National Center for Health Statistics
NHANES: National Health and Nutrition Examination Survey
OR: Odds ratio
PHQ-9: Patient Health Questionnaire
PIR: Poverty income ratio
PPFs: Positive psychosocial factors
RCS: Restricted cubic spline
RF: Random forest
ROC: Receiver operating characteristic
SAD: Seasonal affective disorder
SES: Socioeconomic status
SHAP: SHapley Additive exPlanations
SVM: Support vector machine
WHO: World Health Organization
XGBoost: eXtreme Gradient Boosting.

## Supporting Information

Supporting Information Figure S1. Flowchart of eligible study participants. Figure S2. RCS plots of overall LE8, health behavior, and health factors among four age groups. Figure S3. Interpretable results and visualization for XGBoost model based on SHAP values. Table S1. Metrics for the measurement of CVH based on AHA's LE8 scores for different age groups. Table S2. Baseline information of numerical and categorical depression values with different LE8 classifications. Table S3. Weighed ordinal logistic regression representing relationship between LE8 and elevated depression categories for different ages. Table S4. Demographic and socioeconomic characteristics of study participants with LE8 classifications across the three age groups for adults. Table S5. LE8 conditions and feature distribution for four age-specific groups.
