# Supplementary material for: The Association of Elevated Depression Levels and Life's Essential 8 on Cardiovascular Health With Predicted Machine Learning Models and Interpretations: Evidence From NHANES 2007–2018
Source: Depress Anxiety. 2025 Apr 10;2025:8865176. doi: 10.1155/da/8865176 (PMC12006683; doi:10.1155/da/8865176)
Supplement: Supporting Information — Figure S1. Flowchart of eligible study participants. Figure S2. RCS plots of overall LE8, health behavior, and health factors among four age groups. Figure S3. Interpretable results and visualization for XGBoost model based on SHAP values. Table S1. Metrics for the measurement of CVH based on AHA's LE8 scores for different age groups. Table S2. Baseline information of numerical and categorical depression values with different LE8 classifications. Table S3. Weighed ordinal logistic regression representing relationship between LE8 and elevated depression categories for different ages. Table S4. Demographic and socioeconomic characteristics of study participants with LE8 classifications across the three age groups for adults. Table S5. LE8 conditions and feature distribution for four age-specific groups. [file 8865176.f1.docx]

**The Association of Elevated Depression Levels and Life’s Essential 8 on Cardiovascular Health with Predicted Machine Learning Models and Interpretations: Evidence from NHANES 2007-2018**

Zhixing Wu^1,2#^, Pengyuan Xu^3#^, Yali Zhai^2^, Jinli Mahe^1^, Kai Guo^4^, Wuraola Olawole^5^, Jiahao Zhu^6^, Jin Han^7,8^, Guannan Bai^9*^, Lin Zhang^1,10*^

1. School of Public Health and Preventive Medicine, Monash University, Australia, AU

2. Mailman School of Public Health (Biostatistics Track), Columbia University, United States, USA

3. School of Engineering, Monash University, Australia, AU

4. School of Public Health and Baotou Medical College, Inner Mongolia University of Science & Technology, China, CN

5. School of Nursing, Johns Hopkins University, United States, USA

6. Department of Outpatient Chemotherapy, Harbin Medical University Affiliated Hospital, China, CN

7. Center for Global Health Equity, New York University (Shanghai), China, CN

8. Black Dog Institute, University of New South Wales, Australia, AU

9. Children’s Hospital, Zhejiang University School of Medicine, China, CN

10. Suzhou Industrial Park Monash Research Institute of Science and Technology, Monash University, China, CN

**Supplementary Materials**

**Supplementary Figure 1** Flowchart of eligible study participants;

**Supplementary Figure 2** RCS plots of overall LE8, health behavior and health factors among four age groups;

**Supplementary Figure 3** Interpretable results and visualization for XGBoost model based on SHAP values;

**Supplementary Table 1** Metrics for the measurement of CVH based on AHA’s LE8 scores for different age groups;

**Supplementary Table 2** Baseline information of numerical and categorical depression values with different LE8 classifications; **Supplementary Table 3** Weighed ordinal logistic regression representing relationship between LE8 and elevated depression categories for different ages;

**Supplementary Table 4** Demographic and socioeconomic characteristics of study participants with LE8 classifications across three age groups for adults;

**Supplementary Table 5** LE8 Conditions and Feature Distribution for Four age-specific Groups

**Supplementary Figure 1** Flowchart of eligible study participants


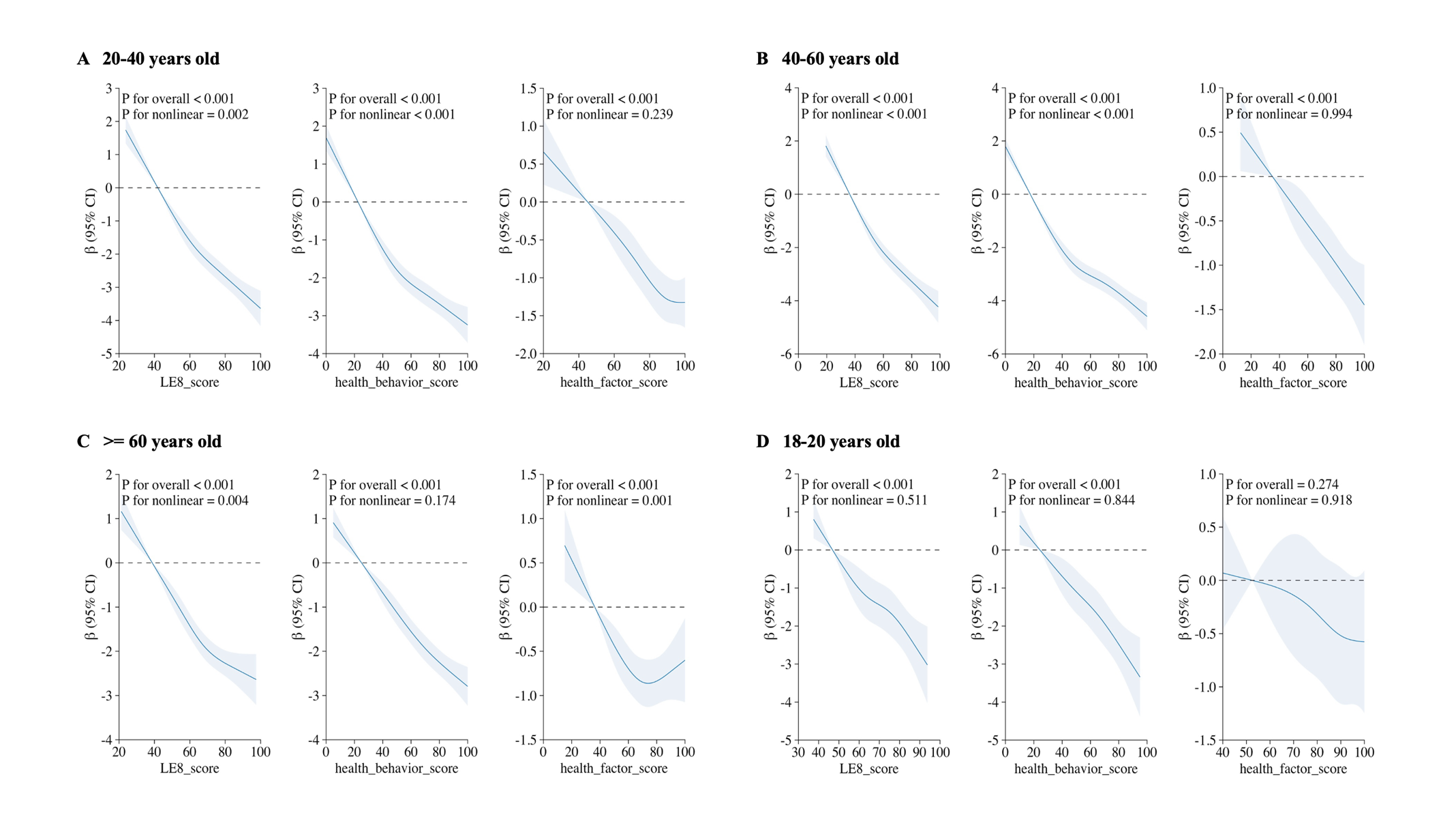


**Supplementary Figure 2** RCS plots of overall LE8, health behavior and health factors among four age groups


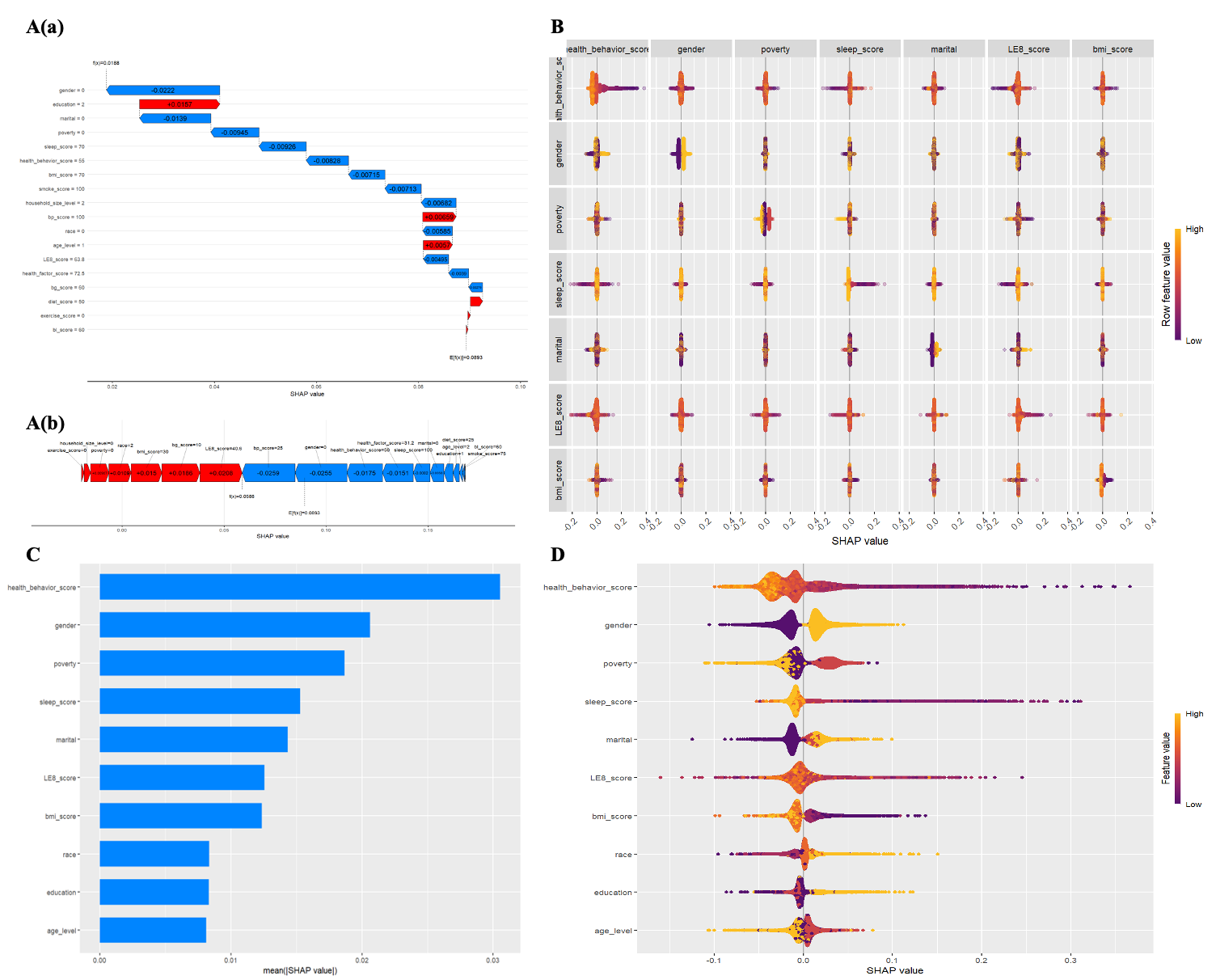


**Supplementary Figure 3** Interpretable results and visualization for XGBoost model based on SHAP values A) Waterfall and SHAP values for different features; B) SHAP value for contributable features; C) Ranking and Feature Importance based on mean SHAP values; D) SHAP summary plot for features and prediction model

**Supplementary Table 1** Metrics for the measurement of CVH based on AHA’s LE8 scores for different age groups

| Domain | CVH metric | Measurement | Quantification of CVH metric (For adults aged ≥ 20 years old) | Quantification of CVH metric (For children aged 2-19 years old) |
| --- | --- | --- | --- | --- |
| Health  Behavior Variables | Diet | Self-reported daily intake of a HEI-2015-style eating pattern | Points Quantile  100 ≥95^th^ (ideal)  80 75 – 94^th^  50 50 – 74^th^  25 25 – 49^th^  0 1 – 24^th^ (least ideal) | Points Quantile  100 ≥95^th^ (ideal)  80 75 – 94^th^  50 50 – 74^th^  25 25 – 49^th^  0 1 – 24^th^ (least ideal) |
|  | Physical  activity | Self-reported minutes of moderate or vigorous physical activity per week | Points Minutes  100 ≥150  90 120 – 149  80 90 – 119  60 60 – 89  40 30 – 59  20 1 – 29  0 0 | Points Minutes  100 ≥420  90 360– 419  80 300 – 359  60 240 – 299  40 120 – 239  20 1 – 119  0 0 |
|  | Nicotine  exposure | Self-reported use of cigarettes or inhaled nicotine-delivery system | Points Categories  100 Never smoker  75 Former smoker, quit ≥5 years  50 Former smoker, quit 1 - <5 years  25 Former smoker, quit <1 year  0 Current smoker  Subtract 20 points (unless score is 0) for living with active indoor smoker in home | Points Categories  100 Never smoker  50 Former smoker, quit ≥1 year  25 Former smoker, quit <1 year & ≥1 month  0 Current smoker  Subtract 20 points (unless score is 0) for living with active indoor smoker in home |
|  | Sleep health | Self-reported average hours of sleep per night | Points Hours  100 7 – <9  90 9 – <10  70 6 – <7  40 5 – <6 or ≥10  20 4 – <5  0 <4 | **Age ≥19 & <20 years old**  Points Hours  100 7 – <9  90 9 – <10  70 6 – <7  40 5 – <6 or ≥10  20 4 – <5  0 <4  **Age <19 years old**  Points Hours  100 8 – <10  90 10 – <11  70 7 – <8  40 6 – <7 or ≥11  20 5 – <6  0 <5 |
| Health  Factor  Variables | Body mass index | Body mass index calculated as weight divided by height squared (kg/m^2^) | Points kg/m^2^  100 <25.0  70 25.0 – 29.9  30 30.0 – 34.9  15 35.0 – 39.9  0 ≥40.0 | Points Level  100 5 – 85^th^ %ile  80 85 – 95^th^ %ile  30 95 %ile – <120% of the 95^th^ %ile  25 120% of the 95^th^ %ile – <140% of the 95^th^ %ile  0 ≥140% of the 95^th^ %ile |
|  | Blood lipids | Non-HDL-cholesterol (calculated from fasting or non-fasting total and HDL cholesterol, mg/dl) | Points mg/dl  100 <130  60 130 – 159  40 160 – 189  20 190 – 219  0 ≥220 | Points mg/dl  100 <100  60 100 – 119  40 120 – 144  20 145 – 189  0 ≥190 |
|  | Blood glucose | Results from casual hemoglobin A1c (%) | Points Categories  100 HbA1c < 5.7%  60 HbA1c 5.7 – 6.3%  40 HbA1c 6.4 – 7.0%  30 HbA1c 7.0 – 7.9 %  20 HbA1c 8.0 – 8.9 %  10 HbA1c 9.0 – 9.9 %  0 HbA1c ≥10.0 % | Points Categories  100 HbA1c < 5.7%  60 HbA1c 5.7 – 6.3%  40 HbA1c 6.4 – 7.0%  30 HbA1c 7.0 – 7.9 %  20 HbA1c 8.0 – 8.9 %  10 HbA1c 9.0 – 9.9 %  0 HbA1c ≥10.0 % |
|  | Blood pressure | Appropriately measured systolic and diastolic blood pressure (mmHg) using appropriately sized blood pressure cuff | Points mmHg  100 <120/<80 (optimal)  75 120 – 129/<80 (elevated)  50 130 – 139 or 80 – 89  25 140 – 159 or 90 – 99  0 ≥160 or ≥100 | Points mmHg  100 <120/<80 (optimal)  75 120 – 129/<80 (elevated)  50 130 – 139 or 80 – 89  25 140 – 159 or 90 – 99  0 ≥160 or ≥100 |

**Note:** This table is adapted and adjusted from Circulation.
**Abbreviation:** LE8, Life’s Essential 8; CVH, Cardiovascular Health; HEI, Healthy Eating Index; HDL, High-density Lipoprotein; AHA, American Heart Association

**Supplementary Table 2** Baseline information of numerical and categorical depression values with different LE8 classifications

| **Characteristics** | **Total** | **Low LE8** | **Moderate LE8** | **High LE8** |
| --- | --- | --- | --- | --- |
| **Age Group: 20-40 years old** | | | | |
| Depression Scores - Mean(95%CI) | 3.13 (2.979, 3.279) | 5.063 (4.556, 5.569) | 3.241 (3.057, 3.425) | 2.169 (1.972, 2.365) |
| Depression Levels - n(%) |  |  |  |  |
| Minimal | 5136 (76.1%) | 483 (59.8%) | 3190 (75.0%) | 1427 (84.6%) |
| Mild | 1052 (15.6%) | 165 (20.5%) | 695 (16.4%) | 205 (12.1%) |
| Moderate | 375 (5.6%) | 102 (12.7%) | 242 (5.7%) | 44 (2.6%) |
| Moderately Severe | 142 (2.1%) | 42 (5.2%) | 97 (2.3%) | 9 (0.5%) |
| Severe | 42 (0.6%) | 15 (1.9%) | 27 (0.6%) | 3 (0.2%) |
| Depression Disease - n(%) |  |  |  |  |
| Yes | 559 (8.3%) | 159 (19.7%) | 366 (8.6%) | 56 (3.3%) |
| No | 6188 (91.7%) | 649 (80.3%) | 3885 (91.4%) | 1632 (96.7%) |
| **Age Group: 40-60 years old** | | | | |
| Depression Scores - Mean(95%CI) | 3.28 (3.097, 3.459) | 5.474 (5.101, 5.847) | 3.072 (2.885, 3.259) | 1.707 (1.472, 1.942) |
| Depression Levels - n(%) |  |  |  |  |
| Minimal | 5370 (75.0%) | 859 (53.7%) | 3505 (77.1%) | 914 (89.7%) |
| Mild | 1133 (15.8%) | 400 (25.0%) | 694 (15.3%) | 83 (8.1%) |
| Moderate | 408 (5.7%) | 214 (13.4%) | 212 (4.7%) | 14 (1.3%) |
| Moderately Severe | 176 (2.5%) | 85 (5.3%) | 95 (2.1%) | 8 (0.8%) |
| Severe | 77 (1.1%) | 41 (2.6%) | 41 (0.9%) | 1 (0.1%) |
| Depression Disease - n(%) |  |  |  |  |
| Yes | 661 (9.2%) | 339 (21.2%) | 348 (7.7%) | 23 (2.2%) |
| No | 6503 (90.8%) | 1259 (78.8%) | 4198 (92.4%) | 997 (97.8%) |
| **Age Group: >= 60 years old** | | | | |
| Depression Scores - Mean(95%CI) | 2.74 (2.572, 2.899) | 4.252 (3.896, 4.609) | 2.592 (2.401, 2.784) | 1.611 (1.293, 1.929) |
| Depression Levels - n(%) |  |  |  |  |
| Minimal | 5837 (79.2%) | 985 (66.1%) | 4097 (80.4%) | 697 (89.4%) |
| Mild | 1056 (14.3%) | 295 (19.8%) | 716 (14.0%) | 72 (9.3%) |
| Moderate | 305 (4.1%) | 141 (9.5%) | 184 (3.6%) | 3 (0.3%) |
| Moderately Severe | 130 (1.8%) | 48 (3.2%) | 79 (1.6%) | 8 (1.0%) |
| Severe | 39 (0.5%) | 22 (1.5%) | 21 (0.4%) | 0 (0.0%) |
| Depression Disease - n(%) |  |  |  |  |
| Yes | 474 (6.4%) | 211 (14.2%) | 284 (5.6%) | 11 (1.4%) |
| No | 6894 (93.6%) | 1280 (85.8%) | 4813 (94.4%) | 769 (98.6%) |
| **Age Group: 18-20 years old** | | | | |
| Depression Scores - Mean(95%CI) | 3.02 (2.679, 3.361) | 4.793 (3.758, 5.828) | 3.368 (2.955, 3.780) | 1.630 (1.299, 1.961) |
| Depression Levels - n(%) |  |  |  |  |
| Minimal | 836 (76.7%) | 43 (51.5%) | 573 (74.1%) | 210 (90.3%) |
| Mild | 185 (17.0%) | 34 (40.2%) | 135 (17.5%) | 22 (9.2%) |
| Moderate | 56 (5.2%) | 5 (5.7%) | 54 (7.0%) | 1 (0.2%) |
| Moderately Severe | 12 (1.1%) | 2 (2.3%) | 10 (1.2%) | 1 (0.3%) |
| Severe | 2 (0.1%) | 0 (0.3%) | 1 (0.2%) | 0 (0.0%) |
| Depression Disease - n(%) |  |  |  |  |
| Yes | 69 (6.4%) | 7 (8.3%) | 65 (8.4%) | 1 (0.5%) |
| No | 1021 (93.6%) | 77 (91.7%) | 708 (91.6%) | 232 (99.5%) |

**Abbreviations:** LE8, Life’s Essential 8; CI, Confidence Intervals

**Supplementary Table 3** Weighed ordinal logistic regression representing relationship between LE8 and elevated depression categories for different ages

| **Age Group: 20-40 years old** | | | | | | | | |
| --- | --- | --- | --- | --- | --- | --- | --- | --- |
|  | **Crude Model** | | | | **New Model** | | | |
|  | **OR** | **Change** | **95% CI** | **p-value** | **OR** | **Change** | **95% CI** | **p-value** |
| **LE8 Category** |  | | | | | | | |
| Low LE8 | 1 (reference) | | | | 1 (reference) | | | |
| Moderate LE8 | 0.464 | -53.6% | (0.377, 0.571) | <0.001 | 0.487 | -51.3% | (0.398, 0.598) | <0.001 |
| High LE8 | 0.250 | -75.0% | (0.185, 0.337) | <0.001 | 0.262 | -73.8% | (0.193, 0.356) | <0.001 |
| **LE8 Scores** |  |  |  |  |  |  |  |  |
| Total Score | 0.972 | -2.9% | (0.966, 0.977) | <0.001 | 0.971 | -2.9% | (0.966, 0.977) | <0.001 |
| Health Behavior Score | 0.977 | -2.3% | (0.973, 0.981) | <0.001 | 0.979 | -2.1% | (0.975, 0.983) | <0.001 |
| Diet | 0.992 | -0.8% | (0.989, 0.996) | <0.001 | 0.994 | -0.6% | (0.991, 0.997) | <0.001 |
| Exercise | 0.994 | -0.6% | (0.993, 0.996) | <0.001 | 0.996 | -0.4% | (0.994, 0.998) | <0.001 |
| Smoke | 0.992 | -0.8% | (0.990, 0.993) | <0.001 | 0.993 | -0.7% | (0.991, 0.994) | <0.001 |
| Sleep | 0.985 | -1.5% | (0.982, 0.988) | <0.001 | 0.986 | -1.4% | (0.984, 0.989) | <0.001 |
| Health Factor Score | 0.994 | -0.6% | (0.990, 0.998) | <0.01 | 0.991 | -0.9% | (0.987, 0.996) | <0.001 |
| BMI | 0.996 | -0.4% | (0.994, 0.999) | <0.01 | 0.996 | -0.4% | (0.994, 0.999) | <0.01 |
| Blood Lipids | 0.999 | -0.1% | (0.996, 1.002) | 0.377 | 0.997 | -0.3% | (0.994, 1.000) | <0.05 |
| Blood Glucose | 0.999 | -0.1% | (0.994, 1.004) | 0.745 | 0.999 | -0.1% | (0.994, 1.005) | 0.765 |
| Blood Pressure | 0.998 | -0.2% | (0.994, 1.002) | 0.291 | 0.995 | -0.5% | (0.991, 0.999) | <0.05 |

| **Age Group: 40-60 years old** | | | | | | | | |
| --- | --- | --- | --- | --- | --- | --- | --- | --- |
|  | **Crude Model** | | | | **New Model** | | | |
|  | **OR** | **Change** | **95% CI** | **p-value** | **OR** | **Change** | **95% CI** | **p-value** |
| **LE8 Category** |  | | | | | | | |
| Low LE8 | 1 (reference) | | | | 1 (reference) | | | |
| Moderate LE8 | 0.337 | -66.3% | (0.286, 0.397) | <0.001 | 0.409 | -59.1% | (0.347, 0.483) | <0.001 |
| High LE8 | 0.130 | -87.0% | (0.096, 0.175) | <0.001 | 0.182 | -81.8% | (0.132, 0.252) | <0.001 |
| **LE8 Scores** |  |  |  |  |  |  |  |  |
| Total Score | 0.956 | -4.4% | (0.952, 0.960) | <0.001 | 0.962 | -3.8% | (0.957, 0.967) | <0.001 |
| Health Behavior Score | 0.967 | -3.3% | (0.963, 0.970) | <0.001 | 0.972 | -2.8% | (0.968, 0.975) | <0.001 |
| Diet | 0.990 | -1.0% | (0.988, 0.992) | <0.001 | 0.992 | -0.8% | (0.990, 0.995) | <0.001 |
| Exercise | 0.989 | -1.1% | (0.987, 0.991) | <0.001 | 0.992 | -0.8% | (0.990, 0.994) | <0.001 |
| Smoke | 0.989 | -1.1% | (0.987, 0.991) | <0.001 | 0.992 | -0.8% | (0.990, 0.994) | <0.001 |
| Sleep | 0.981 | -1.9% | (0.978, 0.984) | <0.001 | 0.984 | -1.6% | (0.981, 0.988) | <0.001 |
| Health Factor Score | 0.985 | -1.5% | (0.980, 0.989) | <0.001 | 0.987 | -1.3% | (0.983, 0.992) | <0.001 |
| BMI | 0.992 | -0.8% | (0.989, 0.994) | <0.001 | 0.993 | -0.7% | (0.990, 0.995) | <0.001 |
| Blood Lipids | 0.995 | -0.5% | (0.992, 0.999) | <0.01 | 0.995 | -0.5% | (0.992, 0.999) | <0.01 |
| Blood Glucose | 0.992 | -0.8% | (0.990, 0.995) | <0.001 | 0.995 | -0.5% | (0.992, 0.997) | <0.001 |
| Blood Pressure | 0.999 | -0.1% | (0.996, 1.001) | 0.252 | 0.999 | -0.1% | (0.997, 1.002) | 0.603 |

| **Age Group: >=60 years old** | | | | | | | | |
| --- | --- | --- | --- | --- | --- | --- | --- | --- |
|  | **Crude Model** | | | | **New Model** | | | |
|  | **OR** | **Change** | **95% CI** | **p-value** | **OR** | **Change** | **95% CI** | **p-value** |
| **LE8 Category** |  | | | | | | | |
| Low LE8 | 1 (reference) | | | | 1 (reference) | | | |
| Moderate LE8 | 0.456 | -54.4% | (0.375, 0.555) | <0.001 | 0.502 | -49.8% | (0.407, 0.619) | <0.001 |
| High LE8 | 0.219 | -78.1% | (0.148, 0.325) | <0.001 | 0.286 | -71.4% | (0.194, 0.422) | <0.001 |
| **LE8 Scores** |  |  |  |  |  |  |  |  |
| Total Score | 0.965 | -3.5% | (0.959, 0.971) | <0.001 | 0.970 | -3.1% | (0.963, 0.976) | <0.001 |
| Health Behavior Score | 0.974 | -2.6% | (0.971, 0.978) | <0.001 | 0.976 | -2.4% | (0.972, 0.980) | <0.001 |
| Diet | 0.993 | -0.7% | (0.991, 0.996) | <0.001 | 0.994 | -0.6% | (0.991, 0.997) | <0.001 |
| Exercise | 0.993 | -0.7% | (0.991, 0.995) | <0.001 | 0.995 | -0.5% | (0.993, 0.997) | <0.001 |
| Smoke | 0.992 | -0.8% | (0.990, 0.994) | <0.001 | 0.992 | -0.8% | (0.990, 0.995) | <0.001 |
| Sleep | 0.981 | -1.9% | (0.978, 0.985) | <0.001 | 0.983 | -1.7% | (0.979, 0.986) | <0.001 |
| Health Factor Score | 0.988 | -1.3% | (0.982, 0.993) | <0.001 | 0.992 | -0.8% | (0.986, 0.999) | <0.05 |
| BMI | 0.993 | -0.7% | (0.990, 0.996) | <0.001 | 0.993 | -0.7% | (0.991, 0.996) | <0.001 |
| Blood Lipids | 0.999 | -0.1% | (0.995, 1.002) | 0.435 | 1.000 | 0.0% | (0.997, 1.004) | 0.810 |
| Blood Glucose | 0.994 | -0.6% | (0.991, 0.998) | <0.001 | 0.996 | -0.4% | (0.992, 0.999) | <0.05 |
| Blood Pressure | 0.999 | -0.1% | (0.997, 1.002) | 0.623 | 1.001 | 0.1% | (0.998, 1.004) | 0.364 |

| **Age Group: 18-20 years old** | | | | | | | | |
| --- | --- | --- | --- | --- | --- | --- | --- | --- |
|  | **Crude Model** | | | | **New Model** | | | |
|  | **OR** | **Change** | **95% CI** | **p-value** | **OR** | **Change** | **95% CI** | **p-value** |
| **LE8 Category** |  | | | | | | | |
| Low LE8 | 1 (reference) | | | | 1 (reference) | | | |
| Moderate LE8 | 0.433 | -56.7% | (0.237, 0.790) | <0.01 | 0.413 | -58.7% | (0.230, 0.739) | <0.01 |
| High LE8 | 0.129 | -87.1% | (0.065, 0.256) | <0.001 | 0.121 | -87.9% | (0.062, 0.235) | <0.001 |
| **LE8 Scores** |  |  |  |  |  |  |  |  |
| Total Score | 0.955 | -4.5% | (0.942, 0.968) | <0.001 | 0.953 | -4.7% | (0.940, 0.966) | <0.001 |
| Health Behavior Score | 0.972 | -2.8% | (0.964, 0.981) | <0.001 | 0.972 | -2.8% | (0.964, 0.981) | <0.001 |
| Diet | 0.988 | -1.2% | (0.980, 0.996) | <0.01 | 0.987 | -1.3% | (0.979, 0.996) | <0.01 |
| Exercise | 0.991 | -0.9% | (0.986, 0.995) | <0.001 | 0.991 | -0.9% | (0.986, 0.996) | <0.001 |
| Smoke | 0.993 | -0.7% | (0.988, 0.998) | <0.01 | 0.992 | -0.8% | (0.987, 0.997) | <0.01 |
| Sleep | 0.991 | -0.9% | (0.985, 0.998) | <0.01 | 0.991 | -0.9% | (0.985, 0.998) | <0.01 |
| Health Factor Score | 0.980 | -2.0% | (0.969, 0.992) | <0.001 | 0.978 | -2.2% | (0.966, 0.990) | <0.001 |
| BMI | 0.991 | -0.9% | (0.985, 0.997) | <0.01 | 0.990 | -1.0% | (0.984, 0.997) | <0.01 |
| Blood Lipids | 0.996 | -0.4% | (0.989, 1.003) | 0.234 | 0.995 | -0.5% | (0.988, 1.002) | 0.163 |
| Blood Glucose | 1.004 | 0.4% | (0.986, 1.023) | 0.657 | 1.005 | 0.5% | (0.986, 1.025) | 0.608 |
| Blood Pressure | 0.988 | -1.2% | (0.976, 1.000) | <0.05 | 0.985 | -1.5% | (0.973, 0.999) | <0.05 |

**Crude Model:** Consider LE8 related variables without further adjustment

**New Model:** Crude model plus all covariates adjusted for gender, gender, education, marital, Poverty-Income ratio and household sizes

**Abbreviations:** LE8, Life’s Essential 8; CI, Confidence Intervals; BMI, Body Mass Index; OR, Odds Ratio

**Supplementary Table 4** Demographic and socioeconomic characteristics of study participants with LE8 classifications across three age groups for adults

| **Age Group: 20-40 years old** | | | | | | | | | |
| --- | --- | --- | --- | --- | --- | --- | --- | --- | --- |
| **Characteristics** | **Total** | | **Low LE8** | | **Moderate LE8** | | **High LE8** | | **p-value** |
| **Sample n** | **6747** | | **808 (11.98%)** | | **4251 (63.01%)** | | **1688 (25.02%)** | |  |
|  | Sample | Pct. | Sample | Pct. | Sample | Pct. | Sample | Pct. |  |
| Gender n(%) |  |  |  |  |  |  |  |  | <0.001 |
| Male | 3502 | 51.9% | 489 | 60.5% | 2345 | 55.2% | 702 | 41.6% |  |
| Female | 3245 | 48.1% | 319 | 39.5% | 1906 | 44.8% | 986 | 58.4% |  |
| Race n(%) |  |  |  |  |  |  |  |  | <0.001 |
| Mexican American | 830 | 12.3% | 122 | 15.1% | 551 | 13.0% | 165 | 9.8% |  |
| Non-Hispanic Black | 783 | 11.6% | 128 | 15.9% | 567 | 13.3% | 105 | 6.2% |  |
| Non-Hispanic White | 4028 | 59.7% | 455 | 56.3% | 2446 | 57.6% | 1110 | 65.8% |  |
| Other | 1107 | 16.4% | 104 | 12.8% | 687 | 16.2% | 307 | 18.2% |  |
| Education n(%) |  |  |  |  |  |  |  |  | <0.001 |
| Less than High School | 877 | 13.0% | 222 | 27.5% | 605 | 14.2% | 81 | 4.8% |  |
| High School or Equivalent | 1430 | 21.2% | 261 | 32.3% | 992 | 23.3% | 210 | 12.5% |  |
| College or Above | 4440 | 65.8% | 325 | 40.3% | 2654 | 62.4% | 1397 | 82.8% |  |
| Marital n(%) |  |  |  |  |  |  |  |  | <0.001 |
| Married | 2719 | 40.3% | 302 | 37.4% | 1712 | 40.3% | 702 | 41.6% |  |
| Never married | 2665 | 39.5% | 242 | 29.9% | 1640 | 38.6% | 759 | 45.0% |  |
| Other | 1363 | 20.2% | 264 | 32.7% | 899 | 21.1% | 226 | 13.4% |  |
| Family PIR n(%) |  |  |  |  |  |  |  |  | <0.001 |
| PIR < 1.3 | 1889 | 28.0% | 334 | 41.3% | 1285 | 30.2% | 309 | 18.3% |  |
| 1.3 <= PIR < 3.5 | 2436 | 36.1% | 309 | 38.2% | 1553 | 36.5% | 581 | 34.4% |  |
| PIR >= 3.5 | 2422 | 35.9% | 166 | 20.5% | 1413 | 33.2% | 798 | 47.3% |  |
| Household Size n(%) |  |  |  |  |  |  |  |  | <0.001 |
| 1-3 member(s) | 2078 | 30.8% | 189 | 23.4% | 1208 | 28.4% | 654 | 38.7% |  |
| 3-5 members | 3056 | 45.3% | 363 | 44.9% | 1964 | 46.2% | 733 | 43.5% |  |
| >=5 members | 1613 | 23.9% | 256 | 31.7% | 1080 | 25.4% | 301 | 17.8% |  |

| **Age Group: 40-60 years old** | | | | | | | | | |
| --- | --- | --- | --- | --- | --- | --- | --- | --- | --- |
| **Characteristics** | **Total** | | **Low LE8** | | **Moderate LE8** | | **High LE8** | | **p-value** |
| **Sample n** | **7164** | | **1598 (22.31%)** | | **4546 (63.46%)** | | **1020 (14.24%)** | |  |
|  | Sample | Pct. | Sample | Pct. | Sample | Pct. | Sample | Pct. |  |
| Gender n(%) |  |  |  |  |  |  |  |  | <0.001 |
| Male | 3446 | 48.1% | 825 | 51.6% | 2267 | 49.9% | 388 | 38.0% |  |
| Female | 3718 | 51.9% | 773 | 48.4% | 2279 | 50.1% | 632 | 62.0% |  |
| Race n(%) |  |  |  |  |  |  |  |  | <0.001 |
| Mexican American | 539 | 7.5% | 113 | 7.1% | 394 | 8.7% | 41 | 4.0% |  |
| Non-Hispanic Black | 744 | 10.4% | 276 | 17.3% | 471 | 10.4% | 33 | 3.2% |  |
| Non-Hispanic White | 4966 | 69.3% | 1020 | 63.8% | 3095 | 68.1% | 810 | 79.5% |  |
| Other | 914 | 12.8% | 189 | 11.8% | 586 | 12.9% | 135 | 13.3% |  |
| Education n(%) |  |  |  |  |  |  |  |  | <0.001 |
| Less than High School | 938 | 13.1% | 362 | 22.6% | 588 | 12.9% | 38 | 3.7% |  |
| High School or Equivalent | 1662 | 23.2% | 557 | 34.8% | 1090 | 24.0% | 88 | 8.6% |  |
| College or Above | 4556 | 63.6% | 680 | 42.5% | 2869 | 63.1% | 894 | 87.7% |  |
| Marital n(%) |  |  |  |  |  |  |  |  | <0.001 |
| Married | 4578 | 63.9% | 815 | 51.0% | 2917 | 64.2% | 780 | 76.4% |  |
| Never married | 658 | 9.2% | 201 | 12.5% | 415 | 9.1% | 60 | 5.9% |  |
| Other | 1929 | 26.9% | 582 | 36.4% | 1215 | 26.7% | 181 | 17.7% |  |
| Family PIR n(%) |  |  |  |  |  |  |  |  | <0.001 |
| PIR < 1.3 | 1275 | 17.8% | 530 | 33.1% | 736 | 16.2% | 75 | 7.3% |  |
| 1.3 <= PIR < 3.5 | 2171 | 30.3% | 590 | 37.0% | 1447 | 31.8% | 186 | 18.2% |  |
| PIR >= 3.5 | 3718 | 51.9% | 478 | 29.9% | 2363 | 52.0% | 760 | 74.5% |  |
| Household Size n(%) |  |  |  |  |  |  |  |  | 0.5 |
| 1-3 member(s) | 3224 | 45.0% | 768 | 48.1% | 1997 | 43.9% | 463 | 45.4% |  |
| 3-5 members | 2794 | 39.0% | 564 | 35.3% | 1823 | 40.1% | 397 | 38.9% |  |
| >=5 members | 1153 | 16.1% | 266 | 16.6% | 726 | 16.0% | 161 | 15.7% |  |

| **Age Group: >= 60 years old** | | | | | | | | | |
| --- | --- | --- | --- | --- | --- | --- | --- | --- | --- |
| **Characteristics** | **Total** | | **Low LE8** | | **Moderate LE8** | | **High LE8** | | **p-value** |
| **Sample n** | **7368** | | **1491 (20.24%)** | | **5097 (69.18%)** | | **780 (10.59%)** | |  |
|  | Sample | Pct. | Sample | Pct. | Sample | Pct. | Sample | Pct. |  |
| Gender n(%) |  |  |  |  |  |  |  |  | 0.3 |
| Male | 3404 | 46.2% | 741 | 49.7% | 2314 | 45.4% | 359 | 46.0% |  |
| Female | 3964 | 53.8% | 750 | 50.3% | 2783 | 54.6% | 421 | 54.0% |  |
| Race n(%) |  |  |  |  |  |  |  |  | <0.001 |
| Mexican American | 287 | 3.9% | 80 | 5.4% | 203 | 4.0% | 13 | 1.7% |  |
| Non-Hispanic Black | 553 | 7.5% | 196 | 13.2% | 368 | 7.2% | 16 | 2.1% |  |
| Non-Hispanic White | 5931 | 80.5% | 1110 | 74.5% | 4088 | 80.2% | 694 | 89.0% |  |
| Other | 598 | 8.1% | 105 | 7.0% | 438 | 8.6% | 56 | 7.2% |  |
| Education n(%) |  |  |  |  |  |  |  |  | <0.001 |
| Less than High School | 1142 | 15.5% | 401 | 26.9% | 763 | 15.0% | 35 | 4.5% |  |
| High School or Equivalent | 1820 | 24.7% | 448 | 30.1% | 1319 | 25.9% | 98 | 12.5% |  |
| College or Above | 4406 | 59.8% | 642 | 43.1% | 3014 | 59.1% | 647 | 83.0% |  |
| Marital n(%) |  |  |  |  |  |  |  |  | <0.001 |
| Married | 4736 | 64.3% | 833 | 55.8% | 3265 | 64.1% | 588 | 75.3% |  |
| Never married | 262 | 3.6% | 79 | 5.3% | 174 | 3.4% | 17 | 2.2% |  |
| Other | 2370 | 32.2% | 580 | 38.9% | 1659 | 32.5% | 175 | 22.5% |  |
| Family PIR n(%) |  |  |  |  |  |  |  |  | <0.001 |
| PIR < 1.3 | 1164 | 15.8% | 433 | 29.1% | 756 | 14.8% | 36 | 4.7% |  |
| 1.3 <= PIR < 3.5 | 2925 | 39.7% | 639 | 42.9% | 2100 | 41.2% | 228 | 29.2% |  |
| PIR >= 3.5 | 3271 | 44.4% | 419 | 28.1% | 2241 | 44.0% | 516 | 66.1% |  |
| Household Size n(%) |  |  |  |  |  |  |  |  | <0.001 |
| 1-3 member(s) | 6003 | 81.5% | 1112 | 74.6% | 4194 | 82.3% | 670 | 85.9% |  |
| 3-5 members | 1062 | 14.4% | 271 | 18.2% | 707 | 13.9% | 97 | 12.5% |  |
| >=5 members | 303 | 4.1% | 109 | 7.3% | 195 | 3.8% | 13 | 1.7% |  |

**Notes**: Marital status labelled as “Other” includes separated, widowed, divorced and living with partner

**Abbreviations**: LE8, Life’s Essential 8; PIR, Poverty-Income Ratio; Pct., Percentage

**Supplementary Table 5** LE8 Conditions and Feature Distribution for Four age-specific Groups

| **Age Groups** | **Total** | | **Low LE8** | | **Moderate LE8** | | **High LE8** | |
| --- | --- | --- | --- | --- | --- | --- | --- | --- |
|  | **Cnt.** | **Pct.** | **Cnt.** | **Pct.** | **Cnt.** | **Pct.** | **Cnt.** | **Pct.** |
| 18-20 years old | 1090 | 4.87% | 84 | 2.11% | 773 | 5.27% | 233 | 6.26% |
| 20-40 years old | 6747 | 30.16% | 808 | 20.30% | 4251 | 28.98% | 1688 | 45.36% |
| 40-60 years old | 7164 | 32.03% | 1598 | 40.14% | 4546 | 30.99% | 1020 | 27.41% |
| >= 60 years old | 7368 | 32.94% | 1491 | 37.45% | 5097 | 34.75% | 780 | 20.96% |
| **Total Population** | **22369** | **100.00%** | **3981** | **100.00%** | **14667** | **100.00%** | **3721** | **100.00%** |

**Abbreviations**: LE8, Life’s Essential 8; Cnt., Count; Pct., Percentage
